# Assessing the potential conditioning effects of mis and disinformation self-efficacy on the relationship between general social media use and political knowledge

**DOI:** 10.3389/fpsyg.2023.1226861

**Published:** 2023-10-31

**Authors:** Toby Hopp, Saima Kazmi

**Affiliations:** Department of Advertising, Public Relations and Media Design, University of Colorado Boulder, Boulder, CO, United States

**Keywords:** political knowledge, social media, self-efficacy, mis and disinformation, news consumption

## Abstract

Prior work on the relationship between general social media use and political knowledge has yielded mixed findings. One recent meta-analysis on the topic concluded that the literature, when assessed as a whole, fails to indicate a direct and statistically identifiable between relationship general social media use and political knowledge. Considering these findings, the present work sought to assess the extent to which general social media use might be conditionall*y* related to political knowledge. To do so, we explored the moderating effect of information-related self-efficacy beliefs. Specifically, building upon general self-efficacy theory and the idea that there exists considerable concern over the extent to which information on social media is factually incorrect, misleading, or biased, we predicted that mis and disinformation self-efficacy (MDSE) beliefs would positively condition the relationship between general social media usage and political knowledge. Contrary to our expectations, the results of three studies indicated that the combination of MDSE and frequent social media use was negatively related to political knowledge.

## 1. Introduction

The ubiquitous presence of social media in modern life raises important questions about the ways in which platforms such Facebook, Twitter/X, and TikTok do and do not influence the contemporary public sphere. As these platforms—and the affordances with which they are associated—continue to remake the political information environment (e.g., Amsalem and Zoizner, 2023), scholars have sought to learn how social media use is connected to political information exposure, consumption, and processing (e.g., Gil de Zúñiga et al., 2017; Oeldorf-Hirsch, 2018; Weeks et al., 2022). Within this broader body of literature, one significant area of emphasis has been on the relationship between social media usage and political knowledge. Highly knowledgeable citizens are increasingly likely to participate in the democratic political process, are comparably likely to possess stable political preferences, and are more likely to resist ideological extremity and incivility (Galston, 2001; Dimitrova et al., 2014; Amsalem and Zoizner, 2023). As such, political knowledge is a fundamental and irreplaceably vital citizen resource in modern democratic societies (Kleinberg and Lau, 2019).

Despite social media’s obvious *potential* to encourage political learning (e.g., Bode, 2016; Boukes, 2019), a recent meta-analysis on the subject concluded that there exists “little evidence that social media contribute to citizens’ knowledge of politics and public affairs. Further testing demonstrates that the overall effect is so close to zero that it cannot be considered theoretically or practically meaningful” (Amsalem and Zoizner, 2023, p. 9). In other words, a systematic summary of the to-date literature provides little, if any, evidence of a consistent and direct relationship between social media usage and the political knowledge outcomes that constitute the lifeblood of democracy.

The literature’s inability to locate a consistent relationship between social media use and political knowledge outcomes may simply be indicative of a baseline reality in which social media usage does not stimulate the political knowledge outcomes long associated with attendance to the mass media (e.g., Chaffee et al., 1994). Given, however, the extent to which social platforms have become an integral part of the modern day information environment and the fact that social media use has been shown to have reliable associations with other democratic outcomes such as political participation (Boulianne, 2016), the proposition that social media is *entirely disconnected* from citizen political knowledge seems to be, frankly speaking, a dubious one. A more probable conclusion is that the fragmented, informationally cluttered, algorithmically influenced, and ever-evolving nature of social media platform usage results in scenarios in which citizen’s social media usage habits are conditionally related to political knowledge outcomes. According to Hayes and Rockwood (2020), conditional effects are those effects that depend upon or vary across “situation, context, stimulus, or individual differences” (p. 20).

One factor that may have meaningful conditioning implications on the relationship between social media use and political knowledge may be the presence of various types of political information that ranges from democratically unhelpful to outright factually incorrect. While some have—probably correctly, in our estimation—observed that social concern over so-called “fake news” has sometimes taken on a morally panicked character (e.g., Farkas and Schou, 2018; Carlson, 2020; Miró-Llinares and Aguerri, 2023), it nonetheless remains the case that large portions of the public has concerns about the extent to which low quality political information has permeated the contemporary epistemic environment. For instance, one recent Pew Research (2022) study indicated that some 70% of Americans see the spread of false information online as a major threat to democracy. Likewise, a cross-national polling effort undertaken by the Reuters Institute indicated that well over half of the study’s respondents were worried about *their own* ability to discern between “real” and “fake” news on the Internet (Newman, 2022). These findings mimic earlier public polling results, which have indicated that most Americans believe that social media-based disinformation has caused widespread confusion about fundamental factual realities (Pew Research Center, 2018). Scholarly work on mis and disinformation suggests that the omnipresent threat (perceived or otherwise) of encountering factually incorrect information when using social media may shape social media user behaviors in several ways. Setting aside the obvious possibility that encountering mis and disinformation can result in the holding of factually incorrect political beliefs, research has, for example, shown that anxiety related to encountering mis and disinformation can cause social media users to disengage with all forms of news and political content encountered on social media (Wenzel, 2019). Another study indicated that perceived exposure to misinformation was associated with information avoidance and a tendency to engage in heuristic (rather than systematic) information processing (Kim et al., 2020). Taken as a whole, these findings suggest that there exists widespread social and user-level concern over the quality of the information encountered on social media and, therein, that this concern has potentially reaching effects on the how and the extent to which users engage with political information on social media.

Building on the foregoing, in the current study, we assess the extent to which information-oriented self-efficacy beliefs may condition the relationship between general social media usage and political knowledge. Self-efficacy describes individual-level judgments pertaining to “how well one can execute courses of action required to deal with prospective situations” (Bandura, 1982). Considered broadly, self-efficacy is a critical individual difference factor that is thought to have widespread effects of motivation, behavior, and social environment (American Psychological Association, 2023). In short, self-efficacy is thought to be relevant to nearly every aspect of human life (Bandura, 1986). In the current research context, our interest lies in exploring the role played by *mis and disinformation self-efficacy* (MDSE; Hopp, 2022). MDSE is a form of epistemic self-efficacy (e.g., Pingree, 2011) that refers to confidence in one’s ability to differentiate between legitimate news and factually incorrect, misleading, and/or hyper-partisan information when using social media platforms such as Facebook (e.g., Khan and Idris, 2019). In other words, MDSE can be understood in an individual’s belief in the extent to which she or he can discern truth in social media information environments. Such capability self-perceptions are critical in light of a wealth of learning-based research showing that individuals with low self-efficacy levels are likely to exhibit amotivation, behavioral avoidance, feelings of information overload, and, ultimately, poor performance-related outcomes.

The current focus on MDSE allows for the examination of a modality-specific epistemic self-efficacy factor that may influence the ability to derive political knowledge from social media use. Perhaps more specifically, we draw upon decades of theorization in human self-efficacy (e.g., Bandura, 1986) to predict that those with heightened levels of MDSE will possess the internal resources necessary to actively and effectively engage in critical social media-based processing, sensemaking, and informational organization behaviors and subsequently, that these capabilities will allow for highly efficacious people to better tap into the political knowledge potentials of social media.

## 2. Literature review

### 2.1. Political knowledge

While prior works have approached “political knowledge” from a number of perspectives (see, for instance, Eveland et al., 2009), the paradigmatic approach in the political and communication sciences has been to conceptualize political knowledge as political information stored in long-term memory. As such, in this study, political knowledge is defined as “the various bits of information about politics that citizens hold” (Carpini et al., 1993, p. 1179).

There exists a widespread consensus among political science and mass media theorists that democratic governance requires a knowledgeable citizenry (Hochschild, 2010; Amsalem and Zoizner, 2023). Indeed, according to Carpini et al. (1993), “Factual knowledge about politics is a critical component of citizenship, one that is essential if citizens are to discern their real interests and take effective advantage of the civic opportunities afforded them” (p. 3). In addition to supporting rational voter behavior and civic engagement, political knowledge is also thought to help mitigate population-level tendencies toward ideological extremism and absolutism, inspire feelings of actor-level political efficacy, and encourage attitudinal stability (e.g., Galston, 2001; Dimitrova et al., 2014; Hopp, 2022; Amsalem and Zoizner, 2023). While some have posited that political knowledge can be understood in various ways (e.g., Eveland and William, 2004), scholars typically conceptualize the construct as the extent to which political facts can be retrieved from long-term memory (Carpini et al., 1993; Suk et al., 2022; Hopp et al., 2023; Martin and Sharma, 2023).

Given its presumed importance to democratic functioning, the topic of political knowledge has received widespread scholarly attention. Despite such research intensity, however, there remain significant knowledge gaps in the ways in which Americans learn about surrounding political realities. In the late pre-Internet age, there existed broad agreement that a primary means by which political information was acquired was through consumption of the traditional mass media (e.g., Chaffee et al., 1994; Chaffee and Kanhihan, 1997; Leshner and McKean, 1997). Since then, however, the political information environment has undergone significant and repeated shocks, shocks that have ultimately resulted in a fundamental re-making of many of the citizen information acquisition routines that emerged and solidified in the post-WW2 era. Central to this epistemic remaking has been the diffusion of social media, and therein, the reality that Americans obtain “a large—and constantly growing—share of their political information through social network sites” (Amsalem and Zoizner, 2023, p. 3).

### 2.2. Social media use and political knowledge

Around 70% of Americans have at least one active social media account, making “social media” a primary means by which information is generated, diffused, and re-mixed in modern society (Pew Research Center, 2021). The literature on social media use has typically understood “social media usage” as directed engagement with some combination of the content and users that collectively constitute a platformed environment (e.g., Boulianne, 2016). In other words, social media usage refers to social media-based content consumption, content engagement, content creation, and engagement with other users.

In contrast to traditional media forms, the information creation and transfer processes and outcomes on social media platforms are shaped by a myriad of intersecting platform features (e.g., algorithmic recommendations, moderation standards) and user factors (e.g., manual feed curation, network composition), resulting in a fragmented “mass interpersonal” (Neubaum and Krämer, 2017) media environment that is defined by the often erratic movement of information. This unevenness has resulted in conflicting theoretical accounts of social media’s potential for political knowledge acquisition. Some scholars (e.g., Woo-Yoo and Gil-de-Zúñiga, 2014; Bode, 2016) have argued that social media can address the so-called political knowledge gap because typical patterns of use can result in an enhanced likelihood of incidental political information exposure. This theoretical rendering is centered on the idea of political information acquisition as a byproduct of social media use: “The basic idea is that news from traditional news media somehow ‘trickle-down’ to users of social media” (Shehata and Strömbäck, 2021). Other theoretical perspectives on centered on the active audience potentialities of social media. Social media platforms allow people to construct networks that pertain to issues and events of interest. Once established, these networks can provide a convenient means of staying abreast of happenings in the surrounding social and political world, thereby offering sufficiently motivated users a straightforward means of acquiring political knowledge (e.g., Park, 2019; Park and de Zúñiga, 2021).

However, as Lee and Xenos (2019) note, “greater opportunities for learning made possible by social media do not necessarily mean that people actually learn as a result of this use” (p. 18). As such, one contrasting theoretical perspective suggests user abilities to filter and hyper-personalize informational inputs can result in selective exposure and informative parametrizing outcomes that are actually detrimental to political knowledge levels (e.g., Aelst et al., 2017; Cacciatore et al., 2018). Still other perspectives suggest that the varied user motivations for using social media, patterns of user information selection and attendance, the composition and behavior of assembled user networks, and other intangible factors result in a scenario where the population level effects of social media use on political information (be they negative or positive) are essentially negligible (e.g., Lee and Xenos, 2019; Amsalem and Zoizner, 2023). Still other perspectives suggest that the combination of information types (interpersonal information, social information, political information, and so on) and information modality (text, picture, video) result in a scenario where social media use exposes users to an “avalanche of information” (van Erkel and Van Aelst, 2021, p. 412). This informational avalanche ultimately stimulates feelings of information overload, which are thought to effectively paralyze learning processes.

Given the foregoing theorizing, it should be of perhaps little surprise that empirical work on the topic of social media use and political knowledge has failed to consistently identify a direct relationship between the two constructs. Amsalem and Zoizner’s (2023) meta-analysis on the subject identified relational effects ranging from *d* = 0.50 [i.e., moderately positive in nature; (Ganduri et al., 2020) to *d* = −0.36 (i.e., moderately negative in nature; Ognyanova, 2022)], indicating a wide variance in terms of both signage and magnitude. In the aggregate, Amsalem and Zoizner’s (2023) meta-analysis found that the average effect apparent in the literature was *d* = 0.02, leading to the conclusion that “on average across studies, respondents, and contexts, social media use has no detectable effect on citizens’ political knowledge” (p. 6). Table 1 provides a snapshot summary of recent examples of work on the subject of social media use and political knowledge. As shown, the effects vary from study to study (in terms of both signage and magnitude) and have a tendency to yield estimates that are not statistically discriminable from zero.

**Table 1:** Examples of prior research findings describing the relationship between social media use and political knowledge.

Study	Findings
Andı et al. (2020)	General social media use negatively and significantly related to political knowledge
Cacciatore et al. (2018)	General Facebook use negatively but not significantly associated with political knowledge; however, Facebook news consumption and news sharing negatively and significantly associated with political knowledge
Dimitrova et al. (2014)	Social media use negatively not significantly related to political learning
Gil de Zúñiga and Goyanes (2021)	General social media use negatively and significantly related to political knowledge
Gil de Zúñiga et al. (2017)	General social media use negatively but not significantly related to political knowledge
Hopp et al. (2020)	General Facebook use negatively and significantly related to political knowledge
Hopp et al. (2023)	General Facebook use negatively but not significantly associated with political knowledge
Kim et al. (2020)	General social media use negatively and significantly related to political knowledge
Martin and Sharma (2023)	General social media use negatively but not significantly related to political knowledge
Park and de Zúñiga (2021)	General social media use negatively and significantly associated with political knowledge
Park (2019)	General social media use negatively but not significantly associated with political knowledge
Park and Kaye (2018)	General social media use positively but not significantly related to political knowledge
Park and Kaye (2019)	General social media use positively but not significantly related to political knowledge
Stephens et al. (2014)	General social media use negatively but not significantly related to political knowledge
Weeks et al. (2022)	Status as a social media user positively but not significantly related to political knowledge

In light of the variance observed in relational estimates between social media use and political knowledge, it may be the case that there exist contingency factors and conditions (Hayes and Rockwood, 2020) that regulate or moderate the relationships between the two variables. As Memon et al. (2019) point out, the assessment of conditioning effects “symbolizes the maturity and sophistication of a field of inquiry” (i). In other words, having ascertained little evidence exists of a consistent, direct, and durable relationship between social media use and political knowledge, the next crucial theoretical step is to assess conditions under which social media and political knowledge might be conditionally related to one another.

As mentioned in this article’s introductory section, there exist reasons to believe that individual user perceptions pertaining to self-possessed abilities to navigate information appearing in social media news feeds may result in dramatically different user-level knowledge-relevant outcomes. Indeed, the prevalence of low-quality information on social media has led some scholars to suggest that we are living in the midst of an *information disorder*, or an epistemic environment in which “trustworthy information is difficult to distinguish from an overwhelming din of competing, and in some cases, conflicting, voices” (Starbird, 2020). Epistemic navigation of disorderly social media environments is largely left to the individual user, who often ends up experiencing feelings of information overload and uncertainty (van Erkel and Van Aelst, 2021). While platforms have developed some practices designed to stem the tide of mis and disinformation, the implementation of these practices has been marked by inconsistency, technical limitations, and other problems that have resulted in a general failure to institute factual guardrails (e.g., Myers and Grant, 2023). And, while there exists a potentially inexhaustible list of individual-level factors that may moderate users’ motivation and ability to derive accurate political information from social media use, we, in this study, focus on issues of self-efficacy, which play a foundational role in human motivation, behavior, and performance (e.g., Bandura, 1982, 1986). Prior to discussing our expectations surrounding the moderating effects of self-efficacy, we first describe this study’s focus on general social media use rather than more targeted forms of social media use, such as purposeful news scanning.

### 2.3. General vs. news-related social media use

As Amsalem and Zoizner (2023) point out, when assessing the relationship between social media use and political knowledge, some prior work has focused on general platform usage (e.g., platform usage intensity, platform usage frequency, platform log-in frequency), while other studies have specifically sought to understand the knowledge impact of purposefully using social media to acquire news-related information. In this particular study, we focus on the knowledge-related effects of general social media use. This decision was made for three reasons. First, perspectives on incidental exposure suggest that one fundamental way that people gain political knowledge in social media settings is through accidental or non-purposeful exposure to news information (e.g., Lee and Kim, 2017; Nanz and Matthes, 2022). Second, and relatedly, while many people report getting the news on social media at least sometimes (60–70% of Americans; Pew Research, 2021), only a fraction of the citizenry reports frequently, substantially, or solely relying on social media for the acquisition of news and news-related information (15–25%; Atske, 2021; Bridge, 2022). This suggests that incidental or otherwise non-purposefully directed news consumption might be the primary means by which political information is ingested on social media platforms. Third, Amsalem and Zoizner’s (2023) meta-analysis failed to find evidence that social media usage purpose (general use vs. use specifically for news-seeking) moderated the relationship between social media use and political knowledge. In light of this finding, we sought, in this study, to focus on social media usage as a general communication behavior.

### 2.4. Mis and disinformation self-efficacy

Self-efficacy refers to internal judgments about one’s ability to successfully undertake a considered course of action (e.g., Bandura, 1977, 1982, 1993). Initially developed as a component of social cognitive theory (Bandura, 1986), self-efficacy currently stands as one of the most frequently utilized theoretical perspectives in social psychology, cognitive psychology, and a wide array of related fields (Hocevar et al., 2014). The widespread application of self-efficacy theory is due, in large part, to its extensive implications for human motivation, affect, cognition, and behavior (Bandura, 1977, 1982, 1993; Bucy and Tao, 2007). Indeed, according to Pajares (2006), “self-efficacy beliefs provide the foundation for motivation, well-being, and personal accomplishment in all areas of life” (p. 339).

Self-efficacy is, inherently, a regulatory mechanism that governs human motivation. In scenarios characterized by self-efficacy deficits, individuals are increasingly likely to pursue strategies of amotivation and action avoidance. That is, when actors do not believe they possess the internal and external resources necessary for successful task completion, they have a marked tendency to re-direct psychological and physical resources to alternate areas of life. Conversely, in situations where efficacy beliefs are high, human agents are substantially more likely to exhibit task-relevant motivation, are substantially more likely to devote cognitive and other psychological resources to the target task, are increasingly likely to demonstrate resiliency, and, ultimately, tend to exhibit higher levels of performance in both the short and long terms (e.g., Bandura, 1977, 1982, 1986, 1994; Pajares, 2006).

Self-efficacy beliefs are not, typically speaking, understood to be enduring context-generic attributes, but are instead linked to distinct realms of functioning (Bandura, 2006). In the context of online information evaluation, prior work has repeatedly shown that sensemaking outcomes are meaningfully tied to self-efficacy levels (e.g., Bucy and Tao, 2007; Hocevar et al., 2014; Khan and Idris, 2019; Hopp, 2022). When people lack the self-perceived capability to actively make sense of encountered information, they are increasingly likely to engage in avoidance behaviors, experience paralyzing feelings information overload, and/or employ non-systematic information processing strategies (e.g., Lo et al., 2013; Schmitt et al., 2018; Hopp, 2022).

Turning directly to the current research topic, we suggest that MDSE may play a crucial role in the ability to epistemically navigate informationally complex social media environments. In line with prior research, we define MDSE as a form of epistemic self-efficacy (Pingree, 2011) that speaks directly to one’s *perceived* ability to make accurate factual assessments of news and news-like information encountered during social media use (Khan and Idris, 2019; Hopp, 2022), and propose that MDSE may conditionally govern the relationship between social media platform use and political knowledge. In situations where MDSE is high, social media users should possess the internal capabilities necessary to attend to, process, and, ultimately, make credibility determinations of encountered information. In this way, the combination of high levels of MDSE and frequent social media use should be associated with heightened levels of political knowledge. Alternately, in situations governed by low levels of MDSE, we predict that the knowledge-oriented potentials of social media use are severely blunted. These expectations are rooted in the theorization described below.

A core feature of the self-efficacy theory is that self-judgments around capability have fundamental implications for motivation (Bandura, 1977, 1982, 1986). When people lack faith in their ability to successfully complete a course of action, they have a pronounced tendency to direct their energies toward other “safer” endeavors. In the context of social media behaviors Boukes (2019) previously noted that some level of user motivated attendance is a key ingredient when it comes to learning from social media. Indeed, prior work by Hong (2006) found that those with high levels of Internet self-efficacy were comparatively more likely to dedicate substantial time and effort to the thorough completion of an information-seeking task. A later study by Cao et al. (2016) found that Internet self-efficacy was a positive predictor of online health-seeking behaviors. Finally, a recent study conducted by Hopp (2022) found that low levels of mis and disinformation self-efficacy were associated with a pronounced amotivation effect insofar as those with low in task-relevant self-efficacy were increasingly likely to refrain from meaningfully participating in a political information classification exercise.

MDSE’s regulatory influence over motivation also has implications for attentional patterns on social media. Social media feeds are complex informational amalgams, essentially allowing users to selectively attend to certain types of information to the detriment of other types of information (e.g., Bode et al., 2017). If an evaluator understands themselves to lack the internal capabilities necessary to epistemically parse news-related claims (i.e., to possess low levels of MDSE), self-efficacy theory predicts (e.g., Themanson and Rosen, 2015) that attentional resources are likely to be directed elsewhere (e.g., toward information about interpersonal others, hedonic information, etc.). And according to the cognitive mediation model (CMC), attention is necessary and critical factor when it comes to learning from the news (Eveland and William, 2001).

The motivational implications of MDSE for political learning also have implications for news elaboration. Along with news attention, news elaboration [typically defined as the systemic processing and schematic assimilation of news and news-related information; (e.g., Oeldorf-Hirsch, 2018)] is understood by the CMC (Eveland and William, 2001) to be essential to individual abilities to extract, store, and recall political information. News elaboration is a resource-intensive state that requires the active allocation of effort by the evaluator (Eveland and William, 2004). In light of the regulatory character of self-efficacy generally and MDSE specifically, it, therefore, holds that those high in MDSE will be increasingly likely to elaborate upon the news information encountered on social media, and, as such, will be comparatively likely to extract political knowledge from general platform use.

Finally, and relatedly, MDSE should help guard against information overload (Schmitt et al., 2018), which has been shown to be a significant inhibitor of political learning on social media (e.g., van Erkel and van Aelst, 2021; Tandoc and Kim, 2022). When in a state of information overload, individuals are unable to integrate encountered stimuli into existing memory and knowledge schemas, and, as a result, are rendered unable to process new information (Pentina and Tarafdar, 2014). Because, however, MDSE establishes the baseline conditions necessary for the allocation of attentional and elaborative resources, it stands to reason that those high in MDSE will be better suited to systematically, efficiently, and effectively make sense of the epistemic conditions that define their social media newsfeeds, and, as such, should be less likely to experience the paralyzing and knowledge-inhibiting effects of information overload.

Taken as a whole, the foregoing theorization can be formally articulated in the following hypothesis statement:

*Hypothesis 1*: MDSE will moderate the relationship between general social media use and political knowledge such that the relationship between the latter two variables will become increasingly positive and strong at heightened levels of MDSE.

### 2.5. Platform differences

Affordance theory suggests that social media platforms are governed by differing technological and social factors and features and, as such, that contemporary digital platforms are associated with unique action potentials (e.g., Hutchby, 2001; Majchrzak et al., 2013). These action potentials can, ultimately, result in differing user outcomes across platform environments. While Amsalem and Zoizner’s (2023) meta-analysis failed to indicate cross-platform differences (Facebook vs. Twitter) regarding political knowledge outcomes, this finding speaks only to the direct relationship between social media use and political knowledge. One platform affordance that may be relevant in the current research context is *information density* (Molyneaux, 2017). Information density refers to the amount of information that is potentially available to an evaluator and can have significant implications for knowledge acquisition (e.g., Eveland and William, 2004). In the present case, we note that some platforms (Facebook, Twitter) allow for a multiplicity of information types (text, still image, video) and feature on/off-platform hyperlinking as a central site functionality. Alternatively, sites like Instagram, TikTok, and YouTube de-emphasize textual information (in favor of still and moving images) and tend to treat hyperlinking capabilities as a periphery platform function. Another platform affordance that may be impactful as it pertains to political learning is *current affairs centricity* (Boukes, 2019), or the extent to which information about current affairs (rather than, for example, information about social others) is explicitly facilitated and encouraged by a platform. Until recently, Twitter, for example, positioned itself as an “information sharing community” (Gleason, 2013, p. 979) that used features such as trending topics to encourage collective discussions. This can be contrasted against Facebook, which has historically prioritized bi-directional social relationships (Boukes, 2019). Considered relative to MDSE, this suggest that there exists *the possibility* that certain platforms might have greater repositories of information that can be taken advantage of by sufficiently motivated users. As such, the following research question is posed:

*Research Question 1*: Does the proposed moderating influence of MDSE on the relationship between general social media platform use and political knowledge vary across platforms?

## 3. Method (primary study)

An online survey was employed. The questionnaire was hosted on the researchers’ institutional Qualtrics server. Participant recruitment was managed by Dynata. A convenience-based quota sample was constructed using the US-population level estimates for age, gender, and educational obtainment. To participate in the study, respondents were required to be current U.S. citizens and 18 years or older. Data was collected in November 2021. A total of 776 complete and valid responses were obtained. Because a series of follow-on studies were conducted (see below), this dataset is hereafter referred to as the “Primary Study” for clarity.

### 3.1. Measures

#### 3.1.1. Political knowledge

Consistent with prior work (e.g., Carpini and Keeter, 1996; Cacciatore et al., 2018; Gil de Zúñiga and Goyanes, 2021; Hopp et al., 2023), political knowledge was measured using eight multiple-choice items. The general goal of this approach was to assess a participant’s ability to recount the *types* political facts typically understood by democratic theorists to be relevant to democratic enactment (Barabas et al., 2014). Moreover, by using an operational approach that mimicked other, prior studies on the relationship between social media and political knowledge (e.g., Cacciatore et al., 2018; Oeldorf-Hirsch, 2018; Kim et al., 2020; Hopp et al., 2023), the results of the current study were directly transferable to the broader body of knowledge on the association between social media use and political knowledge. Because political knowledge encompasses both current events and civics-type topical areas (Barabas et al., 2014), four questions probed participant knowledge of ongoing political events and actors (*who Sonia Sotomoyer is*, *what party currently controls the U.S. House of Representatives*, *who the current U.S. Senate majority leader is*, and *who the current U.S. Secretary of State is*) and four questions assessed static facts about U.S. democracy (*how long a U.S. senator is elected for*, *topical area covered by the 4th Amendment*, number of voting members in the U.S. House of Representatives, and *the number of justices on the U.S. Supreme Court*). As has been done in prior research (e.g., Dimitrova et al., 2014), all questions had a time limit of 20 s. If a response was not provided at the end of the 20-s window, the survey proceeded to the next question. Each question had five response categories, including a “Do not Know” option. Responses were coded as 0 = correct answer not provided and 1 = correct answer provided and summed. “Do not Know” and blank responses were coded as 0.

#### 3.1.2. General social media use

To measure general social media use, respondents were asked to indicate how often they use (1 = never, 7 = frequently) Facebook, Twitter, Instagram, TikTok, and YouTube. The five platforms of central interest to this study were selected because they represent the five most popular social media platforms in the US (Iskiev, 2023).

#### 3.1.3. Mis and disinformation self-efficacy

MDSE was measured using three items, all on seven-point scales where 1 = strongly disagree and 7 = strongly disagree. The indicators were developed using Bandura’s (2006) guidelines for domain-specific self-efficacy scale construction and were consistent with prior, similar empirical studies (Hopp, 2022). Item wording was as follows: (1) *I’m confident in my ability to spot fake news stories on social media*; (2) *I’m confident in my ability to distinguish between fake news and legitimate news stories on social media*; and (3) *I’m confident in my ability to identify inaccurate news content on social media*.

#### 3.1.4. Covariates

The survey instrument also measured a variety of socio-demographic, political, and media use factors. Specifically, in terms of socio-demographic variables, information was collected on age, biological sex, race, educational obtainment (1 = a high school degree or less, 5 = a master’s degree or higher), and estimated annual income (1 = $0.00–$25,000, 7 = Greater than $200,000). For political factors, ideological self-placement (1 = strongly conservative, 11 = strongly liberal), political party identification (democrat, republican, independent, and other party members), and political extremity (the ideological self-placement variable was recoded such that values of 1 and 11 were coded as a 6, values of 2 and 10 were coded as a 5, and so on). News exposure was evaluated by asking participants how frequently they read the newspaper (online or hardcopy), watch broadcast news, watch cable news, read news blogs, and purposefully search for news on social media.

Descriptive statistics for the Primary Study variables are provided in Table 2.

**Table 2:** Descriptive statistics for study variables (Primary Study, Follow-On Studies 1 and 2).

	Primary Study	Follow-On Study 1	Follow-On Study 2
Age	*M* = 46.81, SD = 18.02	*M* = 42.98, SD = 14.76	*M* = 36.56, SD = 10.89
% Female	54%	53%	49%
% White	76%	76%	78%
Income	Median = $50,001–$75,000 [3]	Median = $50,001–$75,000 [3]	Median = $50,001–$75,000 [3]
Education	Median = 2 yr. degree [3]	Median = Some college [3]	Median = 4 yr. degree + [4]
Conservatism	*M* = 6.49, SD = 2.99	*M* = 6.24, SD = 2.90	*M* = 4.71, SD = 3.47
Political extremity	*M* = 3.41, SD = 1.84	*M* = 3.18, SD = 1.92	*M* = 4.37, SD = 1.53
% Dem	36%	39%	63%
% Rep	33%	32%	28%
% Independent/other	31%	29%	9%
Newspaper consumption	*M* = 3.68, SD = 2.19	*M* = 3.62, SD = 2.17	*M* = 5.21, SD = 1.49
Broadcast news consumption	*M* = 4.23, SD = 2.15	*M* = 4.52, SD = 2.08	*M* = 5.17, SD = 1.45
Cable news consumption	*M* = 3.71, SD = 2.20	*M* = 4.05, SD = 2.27	*M* = 5.23, SD = 1.56
Social media news consumption	*M* = 3.32, SD = 2.03	*M* = 3.96, SD = 2.09	*M* = 5.30, SD = 1.43
Facebook use	*M* = 4.63, SD = 2.38	*M* = 4.84, SD = 1.66, *r* = 0.56	*M* = 5.40, SD = 1.47
Twitter use	*M* = 2.59, SD = 2.19	–	*M* = 5.40, SD = 1.47
Instagram use	*M* = 3.26, SD = 2.40	–	*M* = 5.59, SD = 1.42
YouTube use	*M* = 4.37, SD = 2.30	–	*M* = 5.82, SD = 1.14
TikTok use	*M* = 2.92, SD = 2.40	–	*M* = 5.00, SD = 1.15
Mis and disinformation self-efficacy	*M* = 4.78, SD = 1.39, α = 0. 91	M = 4.98, SD = 1.23, α = 0.93	*M* = 5.44, SD = 0.99, α = 0.74
Political knowledge	*M* = 2.92, SD = 2.24, KR-20 = 0.75	M = 1.91, SD = 1.46, KR-20 = 0.71	*M* = 3.81, SD = 2.04, KR-20 = 0.62
*N*	776	1,029	1,214

## 4. Results

The primary hypothesis motivating this study was assessed using a series of ordinary least squares (OLS) regression models. To aid model intepretability, all continuous predictors were mean-centered and standardized prior to model estimation. For the purposes of determining statistical significance, robust standard errors (HC3) were used.

As seen in Table 3 (Model 1), MDSE was positively associated with political knowledge, *b* = 0.33, se = 0.07, *p* < 0.001. Of the general social media usage variables, both the Facebook (*b* = −0.23, se = 0.07, *p* < 0.01) and TikTok (*b* = −0.22, se = 0.08, *p* < 0.01) usage variables were negatively related to political knowledge. We did not observe significant associations between the Twitter, Instagram, or YouTube variables and political knowledge.

**Table 3:** Ordinary least squares models depicting the relationship between general social media platform use, mis and disinformation self-efficacy (MDSE) and political knowledge (Primary Study).

	Model 1	Model 2	Model 3	Model 4	Model 5	Model 6
Age	0.62***	0.63***	0.62***	0.65***	0.64***	0.64***
	(0.10)	(0.10)	(0.10)	(0.10)	(0.10)	(0.10)
Sex (1 = Female)	−0.61***	−0.61***	−0.62***	−0.62***	−0.62***	−0.62***
	(0.15)	(0.15)	(0.15)	(0.15)	(0.15)	(0.15)
Race (1 = White)	0.09	0.08	0.09	0.11	0.10	0.10
	(0.17)	(0.17)	(0.17)	(0.17)	(0.17)	(0.17)
Income	0.07	0.08	0.07	0.07	0.07	0.07
	(0.08)	(0.08)	(0.08)	(0.08)	(0.08)	(0.08)
Education	0.45***	0.45***	0.44***	0.45***	0.44***	0.44***
	(0.08)	(0.08)	(0.08)	(0.08)	(0.08)	(0.08)
Conservatism	−0.10	−0.09	−0.10	−0.11	−0.10	−0.11
	(0.08)	(0.08)	(0.08)	(0.08)	(0.08)	(0.08)
Political extremity	−0.02	−0.02	−0.02	−0.01	−0.02	−0.02
	(0.07)	(0.07)	(0.07)	(0.07)	(0.07)	(0.07)
Dem - rep contrast	0.39*	0.39*	0.38*	0.37*	0.40*	0.39*
	(0.19)	(0.18)	(0.18)	(0.18)	(0.18)	(0.18)
Dem - Ind./other contrast	−0.12	−0.12	−0.12	−0.12	−0.10	−0.12
	(0.17)	(0.17)	(0.17)	(0.17)	(0.17)	(0.17)
Newspaper consumption	0.06	0.08	0.07	0.07	0.06	0.06
	(0.08)	(0.08)	(0.08)	(0.08)	(0.08)	(0.08)
Broadcast news consumption	0.10	0.11	0.10	0.10	0.11	0.10
	(0.09)	(0.09)	(0.09)	(0.09)	(0.09)	(0.09)
Cable news consumption	0.19*	0.19*	0.19*	0.20*	0.19*	0.20*
	(0.09)	(0.09)	(0.09)	(0.09)	(0.09)	(0.09)
Social media news consumption	−0.13	−0.11	−0.13	−0.13	−0.11	−0.12
	(0.10)	(0.10)	(0.10)	(0.10)	(0.10)	(0.10)
Facebook use	−0.23**	−0.26***	−0.24**	−0.24**	−0.25***	−0.23**
	(0.07)	(0.07)	(0.07)	(0.07)	(0.07)	(0.07)
Twitter use	0.15	0.15	0.16	0.16	0.16	0.16
	(0.09)	(0.09)	(0.09)	(0.09)	(0.09)	(0.09)
Instagram use	−0.10	−0.09	−0.10	−0.08	−0.09	−0.08
	(0.09)	(0.09)	(0.09)	(0.09)	(0.09)	(0.09)
YouTube use	−0.15	−0.15	−0.15	−0.15	−0.16*	−0.14
	(0.08)	(0.08)	(0.08)	(0.08)	(0.08)	(0.08)
TikTok use	−0.22**	−0.20*	−0.21*	−0.20*	−0.21*	−0.21*
	(0.08)	(0.08)	(0.08)	(0.08)	(0.08)	(0.08)
MDSE	0.33***	0.31***	0.32***	0.31***	0.32***	0.31***
	(0.07)	(0.07)	(0.07)	(0.07)	(0.07)	(0.07)
Facebook use * MDSE		−0.18**				
		(0.06)				
Twitter use * MDSE			−0.08			
			(0.06)			
Instagram use * MDSE				−0.19**		
				(0.06)		
YouTube use * MDSE					−0.15*	
					(0.06)	
TikTok use * MDSE						−0.16**
						(0.06)
*N*	776	776	776	776	776	776
*R* ^2^	0.38	0.39	0.38	0.39	0.39	0.39

All continuous predictors are mean-centered and scaled by 1 standard deviation. Standard errors are heteroskedasticity robust. ***p < 0.001; **p < 0.01; *p < 0.05.

**Figure 1:**
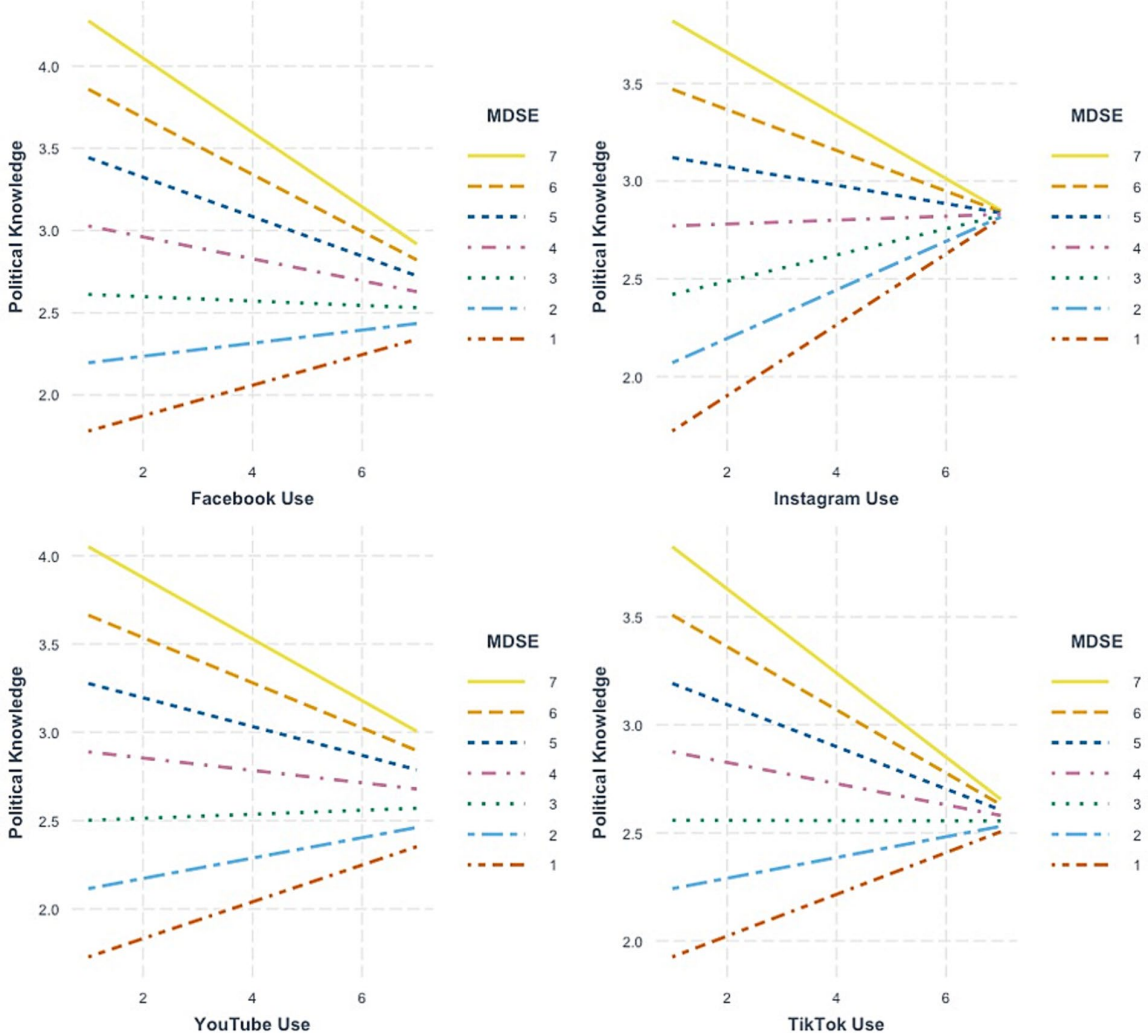
Simple slopes plots depicting the relationship between general social media platform use, mis and disinformation self-efficacy (MDSE) and political knowledge (Primary Study).

Next, as shown in Models 2, 4, 5, and 6 in Table 3, we, as expected, observed significant interaction effects for MDSE and all the general social media usage variables except for Twitter (Facebook * MDSE: *b* = −0.18, se = 0.06, *p* < 0.01; Instagram * MDSE: *b* = −0.19, se = 0.06, *p* < 0.01; YouTube * MDSE: *b* = −0.15, se = 0.06, *p* < 0.05; TikTok * MDSE: *b* = −0.16 se = 0.06, *p* < 0.01). However, when these effects were decomposed, we saw a striking and consistent pattern that ran *contrary* to our hypothesizing. Specifically, as shown in the simple slopes plots presented in Figure 1, we found that high levels of MDSE coupled with frequent social media usage was predictive of *diminished* political knowledge levels. Therein, in scenarios governed by low levels of MDSE, there appeared to be a slight-but-positive relationship between social media usage frequency and political knowledge levels.

Regarding the research question, we did not observe appreciable cross-platform differences. As shown in Table 3; Figure 1, the moderating effect of MDSE on the usage-knowledge link was consistent across platform contexts. The only exception to this rule was the coefficient for the Twitter * MDSE product term, which was not significant (*b* = −0.08, se = 0.06, *p* > 0.05).

## 5. Method (follow-on studies 1 and 2)

Given the surprising nature of the results discussed above, we set out to see if the above-observed effects were replicable. To do so, two additional datasets were employed. The first dataset collected by the research team for a separate observational project (Hopp, 2022). This project had a slightly different theoretical focus and employed different criterion factors; however, because the study was generally interested in cognitive responses to the contemporary information environment, it contained many of the same variables employed in the primary study described above. In contrast to the primary study, however, this dataset only contained information about respondents’ Facebook usage habits. As was the case in the primary study, participant recruitment was managed by Dynata. Population-level estimates extracted from Pew Research’s Core Trends Survey (2018) were used to construct quotas for the US-based Facebook using population in terms of age, education, income, biological sex, and racial/ethnic identification. Data was collected in May of 2020. A total of 1,029 complete and valid responses were obtained. For clarity purposes, this dataset is hereafter referred to as “Follow-On Study 1.” The second dataset used for replication purposes was an original dataset collected from Amazon’s Mechanical Turk. To aid data quality, CloudResearch’s^1^ suite of data quality tools was employed to guard against participation by bots, users who have previously provided inconsistent demographic information, users who have previously provided low-quality data, and users located outside of the US. This data was collected in August 2022. A total of 1,214 complete and valid responses were obtained. This dataset is hereafter referred to as “Follow-On Study 2” for clarity purposes.

### 5.1. Measures

#### 5.1.1. Political knowledge

In Follow-On Study 1, political knowledge was measured using four items (*who Sonia Sotomoyer is*, *what party currently controls the U.S. House of Representatives*, *who the current U.S. Senate majority leader is*, and *who the current U.S. Secretary of State is*). As in the Primary study, all questions had a time limit of 20 s, and incorrect, “do not know” and missing questions were coded as incorrect responses. In Follow-On Study 2, the items, completion processes, and coding processes were identical to those used in the Primary Study.

#### 5.1.2. Mis and disinformation self-efficacy

In Follow-On Study 1, the MDSE items and response categories were identical except for the fact that the word “Facebook” was used instead of the phrase “social media.” In Follow-On Study 2, item wordings and response categories were identical to those employed in the Primary Study.

#### 5.1.3. General social media use

In Follow-On Study 1, Facebook usage frequency was assessed by *asking respondents how often they log into Facebook* and *how often they post content on Facebook* (1 = very infrequently, 7 = very frequently). These items were subsequently collapsed into a single composite index. In Follow-On Study 2, respondents were asked to report how frequently they use Facebook, Twitter, Instagram, TikTok, and YouTube (1 = never, 7 = frequently).

#### 5.1.4. Covariates

Covariate measure wordings and measurement procedures in both Follow-On Study 1 and Follow-On Study 2 were identical to those used in the Primary Study, with one exception. The education measure used in Follow-On Study 1 had 6 (rather than 5) response options, ranging from 1 = Less than a High School degree to 6 = A Master’s Degree or Higher.

Descriptive statistics for the variables used in both follow-on studies are provided in Table 2.

## 6. Results

OLS was again used to assess the combinatory effects of social media use and MDSE on political knowledge. All continuous predictors were mean centered and standardized prior to model estimation. Robust standard errors (HC3) were employed.

**Figure 2:**
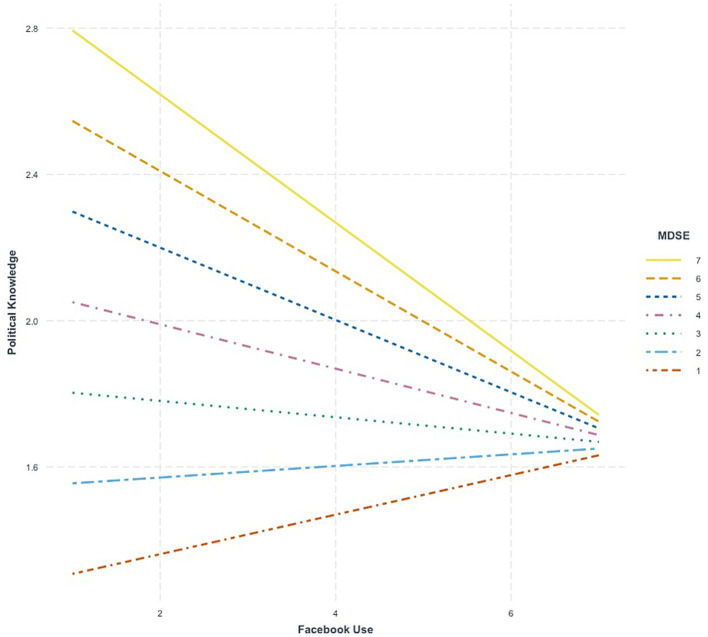
Simple slopes plots depicting the relationship between general Facebook use, mis and disinformation self-efficacy (MDSE) and political knowledge (Follow-On Study 1).

In Follow-On Study 1 (Table 4, Model 1), significant main effects for both MDSE (*b* = 0.13, se = 0.04, *p* < 0.01) and general Facebook use (*b* = −0.16, se = 0.04, *p* < 0.001) were observed. The interaction terms comprised of the two variables was negatively and significantly associated with political knowledge, *b* = −0.08, se = 0.04, *p* < 0.05 (Table 4, Model 2). Decomposition of the interaction effect (Figure 2) shows the same pattern observed in the primary study, namely that at high levels of MDSE, the relationship between general Facebook use and political knowledge is negative at low levels of MDSE, the relationship between general Facebook use and political knowledge becomes marginally positive.

**Table 4:** Ordinary least squares models depicting the relationship between general Facebook use, mis- and dis-information self-efficacy (MDSE) and political knowledge (Follow-On Study 1).

	Model 1	Model 2
Age	0.44***	0.43***
	(0.04)	(0.04)
Sex (1 = Female)	−0.42***	−0.42***
	(0.08)	(0.08)
Race (1 = White)	0.07	0.08
	(0.10)	(0.10)
Income	0.01	0.01
	(0.05)	(0.05)
Education	0.28***	0.28***
	(0.04)	(0.04)
Conservatism	−0.07	−0.07
	(0.05)	(0.05)
Political extremity	0.07	0.08*
	(0.04)	(0.04)
Dem - rep contrast	0.04	0.04
	(0.11)	(0.11)
Dem - Ind./other contrast	−0.23*	−0.22*
	(0.11)	(0.11)
Newspaper consumption	0.09*	0.09*
	(0.05)	(0.05)
Broadcast news consumption	0.01	0.02
	(0.05)	(0.05)
Cable news consumption	0.12*	0.12*
	(0.05)	(0.05)
Social media news consumption	−0.05	−0.05
	(0.05)	(0.05)
Facebook use	−0.16***	−0.16***
	(0.04)	(0.04)
MDSE	0.13**	0.13**
	(0.04)	(0.04)
Facebook use * MDSE		−0.08*
		(0.04)
*N*	1,029	1,029
*R* ^2^	0.31	0.32

All continuous predictors are mean-centered and scaled by 1 standard deviation. Standard errors are heteroskedasticity robust. ***p < 0.001; **p < 0.01; *p < 0.05.

**Figure 3:**
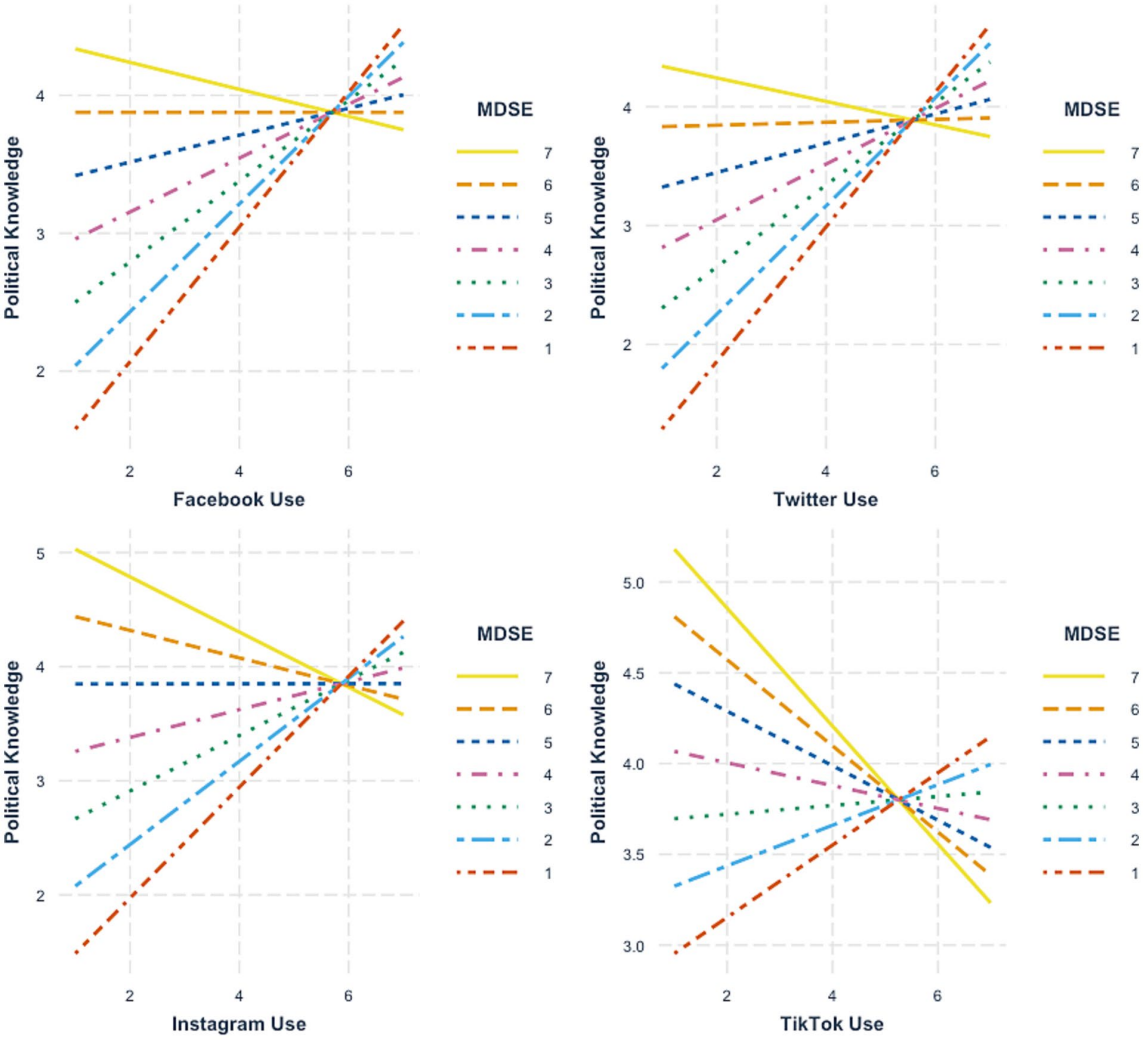
Simple slopes plots depicting the relationship between general social media platform use, mis and disinformation self-efficacy (MDSE) and political knowledge (Follow-On Study 2).

In Follow-On Study 2, we failed to replicate the main effects of MDSE on political knowledge (Table 5, Model 1). Moreover, in terms of the general platform usage variables, only TikTok use was significantly related to political knowledge, *b* = −0.34, se = 0.07, *p* < 0.001. However, looking at the interaction terms (Table 5, Models 2, 3, 4, 6), we again found that MDSE moderated the relationship between political knowledge and Facebook use (*b* = −0.14, se = 0.04, *p* < 0.001), Twitter use (*b* = −0.16, se = 0.05, *p* < 0.01), Instagram use (*b* = −0.17, se = 0.05, *p* < 0.001) and TikTok use (*b* = −0.16, se = 0.05, *p* < 0.05). Examination of the simple slope plots revealed a familiar pattern. Specifically, as shown in Figure 3, we again found that the combination of high levels of general social media use and high levels of MDSE was associated with diminished political knowledge levels. However, when MDSE was low, frequent platform usage was positively associated with political knowledge.

**Table 5:** Ordinary least squares models depicting the relationship between general social media platform use, mis and disinformation self-efficacy (MDSE) and political knowledge (Follow-On Study 2).

	Model 1	Model 2	Model 3	Model 4	Model 5	Model 6
Age	0.02	0.03	0.03	0.03	0.03	0.03
	(0.06)	(0.06)	(0.06)	(0.06)	(0.06)	(0.06)
Sex (1 = Female)	−0.42***	−0.40***	−0.39***	−0.40***	−0.40***	−0.42***
	(0.12)	(0.12)	(0.12)	(0.12)	(0.12)	(0.12)
Race (1 = White)	0.28*	0.25	0.25	0.25	0.27	0.26
	(0.14)	(0.14)	(0.14)	(0.14)	(0.14)	(0.14)
Income	0.02	0.03	0.03	0.03	0.03	0.02
	(0.06)	(0.06)	(0.06)	(0.06)	(0.06)	(0.06)
Education	0.12*	0.13*	0.13*	0.13*	0.12*	0.13*
	(0.06)	(0.06)	(0.06)	(0.06)	(0.06)	(0.06)
Conservatism	0.19**	0.18**	0.18**	0.18**	0.18**	0.18**
	(0.06)	(0.06)	(0.06)	(0.06)	(0.06)	(0.06)
Political extremity	0.09	0.10	0.11	0.10	0.10	0.10
	(0.07)	(0.07)	(0.07)	(0.07)	(0.07)	(0.07)
Dem - rep contrast	−0.76***	−0.75***	−0.74***	−0.74***	−0.75***	−0.74***
	(0.13)	(0.13)	(0.13)	(0.13)	(0.13)	(0.13)
Dem - Ind./other contrast	−0.38	−0.31	−0.32	−0.32	−0.39	−0.33
	(0.23)	(0.23)	(0.23)	(0.23)	(0.23)	(0.23)
Newspaper consumption	−0.10	−0.12	−0.11	−0.13	−0.11	−0.11
	(0.08)	(0.08)	(0.08)	(0.08)	(0.08)	(0.08)
Broadcast news consumption	0.06	0.06	0.06	0.07	0.07	0.07
	(0.08)	(0.08)	(0.08)	(0.08)	(0.08)	(0.08)
Cable news consumption	−0.01	0.00	−0.01	0.00	−0.01	0.01
	(0.09)	(0.09)	(0.09)	(0.09)	(0.09)	(0.09)
Social media news consumption	−0.16*	−0.15*	−0.16*	−0.16*	−0.16*	−0.16*
	(0.08)	(0.08)	(0.08)	(0.08)	(0.08)	(0.08)
Facebook use	0.11	0.08	0.09	0.10	0.10	0.13
	(0.08)	(0.08)	(0.08)	(0.08)	(0.08)	(0.08)
Twitter use	0.14	0.12	0.11	0.12	0.13	0.12
	(0.08)	(0.08)	(0.08)	(0.08)	(0.08)	(0.08)
Instagram use	−0.05	−0.06	−0.06	−0.08	−0.06	−0.05
	(0.08)	(0.08)	(0.08)	(0.08)	(0.08)	(0.08)
YouTube use	0.01	−0.01	0.00	−0.01	−0.02	0.00
	(0.07)	(0.07)	(0.07)	(0.07)	(0.07)	(0.07)
Tiktok use	−0.34***	−0.31***	−0.32***	−0.31***	−0.33***	−0.35***
	(0.07)	(0.07)	(0.07)	(0.07)	(0.07)	(0.07)
MDSE	0.05	0.03	0.02	0.03	0.06	0.02
	(0.07)	(0.07)	(0.07)	(0.07)	(0.07)	(0.07)
Facebook use * MDSE		−0.14***				
		(0.04)				
Twitter use * MDSE			−0.16**			
			(0.05)			
Instagram use * MDSE				−0.17***		
				(0.05)		
YouTube use * MDSE					−0.10	
					(0.05)	
TikTok use * MDSE						−0.16**
						(0.05)
*N*	1,214	1,214	1,214	1,214	1,214	1,214
*R* ^2^	0.09	0.10	0.10	0.10	0.10	0.10

All continuous predictors are mean-centered and scaled by 1 standard deviation. Standard errors are heteroskedasticity robust. ***p < 0.001; **p < 0.01; *p < 0.05.

Finally, in terms of cross-platform differences, the data from Follow-On Study 2 again failed to indicate strikingly different relational patterns from platform-to-platform. Specifically, as seen in Table 5; Figure 3, the moderating effect of MDSE took on a consistent character across the surveyed platforms.

## 7. Discussion

This study proposed that MDSE would positively moderate the relationship between general social media platform use and political knowledge. Perhaps more specifically, we expected those high in MDSE would possess the internal capabilities to derive political knowledge from frequent social media use. The results of the primary study and two follow-on studies provided consistent evidence against our hypothesis insofar as we found that the combination of frequent social media use and high levels of MDSE was associated with *lower* levels of political knowledge. Several possible explanations for this finding are explored in the space below.

The first possible explanation may be linked to the so-called *overconfidence effect* (e.g., Stone, 1994; Moores and Chang, 2009; Fuchs et al., 2019). As Moores and Chang (2009) put it, “there is evidence to suggest that one can become overconfident when a person’s belief about their expected level of performance exceeds their actual performance,” and, therein, that “self-efficacy is only satisfaction in one’s level of performance, and complacency may result, leading to a negative relationship between self-efficacy and performance” (p. 69). Indeed, even Bandura (1997) left open the possibility that in prescribed circumstances, self-efficacy can stimulate feelings of over-confidence, which, in turn, can lead to negative or undesirable performance outcomes. In the context of social media use, it may therefore be the case that MDSE is associated with a false impression that one is well-suited for and well-capable of making accurate epistemic judgments and may, as a result, lead to a lack of external fact-checking, a reliance on one’s own beliefs and feelings as “facts,” and/or an overreliance on affective or otherwise non-systematic judgment mechanisms (i.e., heuristic processing). MDSE overconfidence effects may be especially pronounced in a media and political climate where professional journalism and its methods have been subject to ideological attacks, de-professionalization, and rapid workforce reductions. In short, professional journalism’s dislocation from the public sphere’s epistemic center has been accomplished through a variety of means, many of which have—purposefully or otherwise—undermined the public notion of journalistic practice as rigorous, trustworthy, and, perhaps most importantly, something that requires a skillset beyond what is possessed by the average citizen.

A second explanation may be that high MDSE social media users are, in fact, consuming, processing, and making sense of news-related information on social media, but that the types of political information that appear on these platforms, while not outright disinformation or otherwise factually untrue, differs from the types of political information typically understood as important to democratic enactment (e.g., Barabas et al., 2014). In other words, while social media feeds frequently contain to links to traditional news media sites and also links to newer, web-based platforms that report on democratic structures, government performance, and policy proposals (e.g., www.cookpolitical.com, www.boltsmag.org, www.opensecrets.com), it also has a tendency to amplify information on personal scandal, horse race coverage, and so on. As the American citizenry continues to make an affective turn (Papacharissi, 2015), such information might be understood by citizens as centrally informative to civic and political orientations and behaviors, and, therefore receive enhanced attention. A related explanation might be that the quality of the political information found in users’ social media feeds is of such characteristically low quality that cognitive evaluation processes, ultimately, culminate in democratic knowledge deficits. Typical approaches to news exposure and political learning (e.g., Eveland and William, 2001) hold that elaborating upon evaluated content drives internal processes important to informational classification and storage in long-term memory. These approaches, however, take for granted the information being assessed (i.e., “the news”) bears a factual resemblance to reality. The currently presented results could be an indicator of a “bad path” outcome in which high MDSE social media users are allocating significant energies on processing factually dubious or otherwise low-quality political information, integrating this information into long-term memory, and, ultimately, building internal knowledge structures that are essentially non-factual.

All of that being said, the present results are not totally pessimistic with regard to MDSE. In two of the three samples (Primary Study and Follow-On Study 1), we found a positive and statistically significant direct association between MDSE and political knowledge. Perhaps more importantly, all three samples provided evidence that infrequent social media users with high levels of MDSE demonstrated enhanced political knowledge levels. This finding might suggest that—at least for those with significant domain-relevant self-efficacy resources—moderate levels of social media use can augment other forms of political information consumption and, as such, be a positive contributor to knowledge levels. To some extent, this line of theorizing corresponds with the displacement effect perspective (e.g., Cacciatore et al., 2018), which holds that the use of social media for news consumption is “replacing” other, more traditional forms of mass media-based news consumption. The effects of this replacement have, for the reasons outlined in this study’s literature review, troubled scholars. Our findings suggest that the combination of high levels of MDSE and low-to-moderate levels of social media use may result in a complementary knowledge effect. Presumably, this is because high MDSE users have the cognitive resources necessary to critically evaluate information extracted from a variety of informational sources and formats and, subsequently, integrate this information into long-term memory.

Concerning the direct relationship between social media use and political knowledge, the present data corresponds with prior work (Amsalem and Zoizner, 2023) insofar as it generally fails to locate a consistent effect between the two variables. As shown in Tables 3–5, we saw a divergence in both signage and magnitude between the social media use variables and political knowledge across the three samples. This finding substantiates our study’s warrant (i.e., that there exists an uncertain relationship between social media behaviors and political knowledge outcomes) and underscores the future need for more research on factors that condition (e.g., Hayes and Rockwood, 2020) the relationship between social media usage and political knowledge.

Finally, regarding the research question, we did not find that MDSE’s interactive effects on the social media-political knowledge link substantially diverged across platforms. This finding suggests that while platform affordances may yield different action potentials (Hutchby, 2001; Majchrzak et al., 2013), these action potentials do not manifest in a way that is relevant to MDSE’s knowledge conditioning effects. As noted above, however, we do note that the direct relationship between the type of platform usage and political knowledge did vary somewhat substantially. A glance at the data reveals that TikTok was, for instance, negatively and robustly associated with political knowledge in both the Primary Study and Follow-On Study 2, indicating that TikTok’s particular set of affordances may not be conducive to political knowledge obtainment.

### 7.1. Limitations and future research

This study is marked by a handful of limitations. First, the cross-sectional nature of the data presents obvious limitations with regard to causal inference, and, therein, we cannot rule out the possibility that our results are influenced by the omission of one or more key variables. Second, this study did not use representative samples and, despite the implementation of numerous data-quality measures, the sample used in Follow-On Study 2 was perhaps especially weak, as it did not (in contrast to the Primary Study and Follow-On Study 1) employ population-level quota screens. Third, for the reasons articulated above, we focused on general social media use rather than the specific use of social media for active political news surveillance. Relatedly, there exist potential operational limitations around the present study’s conceptualization of political knowledge. Although the present study used a normative approach when measuring respondent political knowledge levels, it could well be the case that social media use is associated with political knowledge gains that depart from the types of information typically prioritized by democratic theorists. In other words, the types of political knowledge acquired via social media use might not pertain to things such as the partisan distribution in congress or the makeup of the presidential cabinet, but, instead, to political scandals or polling estimates.

Despite the above limitations, and even though we did not confirm our incoming hypotheses, the present work provides evidence that MDSE has potential implications for the conditions under which social media users do and do not glean political knowledge from platform usage. Looking toward future research, the literature might benefit from a close examination of the extent to which MDSE represents a meaningful and motivated capability to assess complex information environments (e.g., social media feeds) or, alternately, if it is simply a marker of citizen overconfidence in their ability to make accurate epistemic judgments. To explore this, researchers might first measure MDSE levels and, subsequently, expose participants to an information environment populated by information of varying credibility. If MDSE is, in fact, an overconfidence marker, there should be a non-existent relationship between observed information sorting outcomes and MDSE. Researchers might also assess the extent to which MDSE is related to general and context-specific forms of overconfidence. If MDSE is a marker of overconfidence, we would expect to see substantial and systematic associations between MDSE and various overconfidence factors. A second area that might be explored is the type of political information gleaned from social media use. This study, like many before it, prioritized a specific type of political information due to longstanding beliefs that such information holds pronounced democratic salience. However, and given social media’s tendency to accentuate human drama and conflict (e.g., Sahly et al., 2019), it may be the case that users are gaining alternate types of information about the political sphere. Future research should comparatively probe the various types of learning that occurs on social media, and subsequently, assess the independent and conjoint effects of such knowledge acquisition on democratic beliefs, attitudes, and behaviors. Other recommendations for future research include replicating the current findings in a nationally representative sample, examining the relationships presented her in a causal context, further exploring the antecedents and consequences of MDSE, and identification of other cognitive and environmental factors that may help explain the inconsistent relationship between social media use and political knowledge outcomes.

## Data availability statement

The raw data supporting the conclusions of this article will be made available by the authors, without undue reservation.

## Ethics statement

The studies involving humans were approved by University of Colorado Boulder Institutional Review Board. The studies were conducted in accordance with the local legislation and institutional requirements. The participants provided their written informed consent to participate in this study.

## Author contributions

TH conceived and designed the study, performed the statistical analysis, and wrote the first draft of the manuscript. TH and SK wrote sections of the manuscript. All authors contributed to the article and approved the submitted version.

## Footnotes

^1^https://www.cloudresearch.com/
